# VSS: variance-stabilized signals for sequencing-based genomic signals

**DOI:** 10.1093/bioinformatics/btab457

**Published:** 2021-06-24

**Authors:** Faezeh Bayat, Maxwell Libbrecht

**Affiliations:** Department of Computing Science, Simon Fraser University, Burnaby, BC V5A 1S6, Canada; Department of Computing Science, Simon Fraser University, Burnaby, BC V5A 1S6, Canada

## Abstract

**Motivation:**

A sequencing-based genomic assay such as ChIP-seq outputs a real-valued signal for each position in the genome that measures the strength of activity at that position. Most genomic signals lack the property of variance stabilization. That is, a difference between 0 and 100 reads usually has a very different statistical importance from a difference between 1000 and 1100 reads. A statistical model such as a negative binomial distribution can account for this pattern, but learning these models is computationally challenging. Therefore, many applications—including imputation and segmentation and genome annotation (SAGA)—instead use Gaussian models and use a transformation such as log or inverse hyperbolic sine (asinh) to stabilize variance.

**Results:**

We show here that existing transformations do not fully stabilize variance in genomic datasets. To solve this issue, we propose VSS, a method that produces variance-stabilized signals for sequencing-based genomic signals. VSS learns the empirical relationship between the mean and variance of a given signal dataset and produces transformed signals that normalize for this dependence. We show that VSS successfully stabilizes variance and that doing so improves downstream applications such as SAGA. VSS will eliminate the need for downstream methods to implement complex mean–variance relationship models, and will enable genomic signals to be easily understood by eye.

**Availability and implementation:**

https://github.com/faezeh-bayat/VSS

**Supplementary information:**

Supplementary data are available at *Bioinformatics* online.

## 1 Introduction

Sequencing-based assays can measure many types of genomic biochemical activity, including transcription factor (TF) binding, histone modifications and chromatin accessibility. These assays work by extracting DNA fragments from a sample that exhibit the desired type of activity, sequencing the fragments to produce sequencing reads and mapping each read to the genome. Each of these assays produces a genomic signal—i.e. a signal that has a value for each base pair in the genome. Examples include ChIP-seq measurements of TF binding or histone modification and measurements of chromatin accessibility from DNase-seq, FAIRE-seq or ATAC-seq. The natural unit of sequencing-based assays is the read count: the number of reads that mapped to a given position in the genome (after extending and shifting; see Section 2).

Read counts of genomic assays have a nonuniform mean–variance relationship, meaning that variance of the data is a function of the read counts, resulting in higher variance for higher read counts and lower variance for lower read counts, which poses a challenge to their analysis. This property means that, e.g. the difference in read count between biosamples is a poor measure of the difference in activity. For instance, a locus having 100 reads in one replicate while 0 in the other is usually considered more significant than a locus with 1100 reads in one replicate and 1000 reads in the other one.

To handle this issue, most statistical models of genomic signals—such as those used in peak calling—model the mean–variance relationship of read counts explicitly using, e.g. a negative binomial distribution (Anders and Huber, 2010; Gierliński *et al.*, 2015; Guo *et al.*, 2012; Hafemeister and Satija, 2019; Harmanci *et al.*, 2014; Love *et al.*, 2014; Rashid *et al.*, 2011; Ren and Kuan, 2019; Whitaker, 1914; Xing *et al.*, 2012; Zhang *et al.*, 2008).

However, negative binomial models are challenging to implement and optimize, so many methods resort to Gaussian models. Two prominent examples include segmentation and genome annotation (SAGA) methods, such as Segway or IDEAS (Chan *et al.*, 2018; Hoffman *et al.*, 2012a, 2012b; Zhang and Hardison, 2017; Zhang *et al.*, 2016), and imputation methods such as ChromImpute, PREDICTD and Avocado (Durham *et al.*, 2018; Ernst and Kellis, 2015; Schreiber *et al.*, 2018). In the former example, many SAGA methods use a Gaussian distribution to model the distribution of genomic signals given a certain annotation label (others binarize signal (Ernst and Kellis, 2012) or use a negative binomial read count model (Mammana and Chung, 2015)). In the latter example, imputation methods optimize a mean squared error (MSE) objective function, which is equivalent to log likelihood in a Gaussian model. More generally, many other analyses use MSE to quantify the performance of the functional genomic analysis, such as those that predict TF binding sites from sequencing data. All such methods suffer from the issue of a nonuniform mean–variance relationship.

Most Gaussian-based methods employ a variance-stabilizing transformation to handle the nonuniform mean–variance relationship. They most commonly use the log or inverse hyperbolic sine transformations (asinh), which have the formulae log(x+c) for a constant *c* (usually 1) and $\text{asinh}(x)=\text{log}(x+\sqrt{x^{2}+1})$, respectively (Huber *et al.*, 2002). (Note that in some cases users may use transformations for purposes other than variance stabilization, such as the use of log to measure order of magnitude.)

Variance-stabilizing transformations can also be beneficial for visualizing genomic signals. Note that Euclidean distance in a 2D plot corresponds to the log likelihood of difference in a Gaussian model, so a nonuniform mean–variance relationship complicates visualization. Although researchers sometimes visualize raw signals, doing so requires carefully choosing a maximum viewing range which is akin to a crude linear + flat transformation, as otherwise the viewing range is dominated by a few outliers. For example, the UCSC genome browser’s default built-in H3K27ac track by default has a maximum viewing range of 100, whereas the track’s maximum value is 3851.

Despite the widespread use of log and asinh transformations to stabilize variance, to our knowledge, no work has evaluated whether they in fact do so. The use of these transformations assumes that the signals have a specific mean–variance relationship (Section 2). Here, we show that, for many genomic signals, this assumption is violated and thus existing transformations do not fully stabilize variance (Section 3). To solve this issue, we present VSS, a method that produces variance-stabilized genomic signals. VSS determines the empirical mean–variance relationship of a genomic signal by comparing replicates. It uses this empirical mean–variance relationship to produce a transformation function that precisely stabilizes variance.

### 1.1 Related work

Three methods have been developed to correct biases in sequencing-based genomic signals. First, fold enrichment (FE) measures a genomic signal as the ratio of reads of the experiment to a control (such as ChIP Input) (Hoffman *et al.*, 2012b). Second, Poisson p-value measures a signal as the log p-value of a Poisson distribution test with a null hypothesis derived from a control distribution (Kundaje *et al.*, 2015). Third, S3norm (Xiang *et al.*, 2020) normalizes a collection of datasets by matching their empirical sequencing depth and signal–noise ratio. However, none of these methods stabilizes the variance of the data (Section 3).

Many of the challenges mentioned here also exist for assays of gene expression such as RNA-seq data (Anders and Huber, 2010; Anders *et al.*, 2013; Bullard *et al.*, 2010; Conesa *et al.*, 2016; Hansen *et al.*, 2012; Huber *et al.*, 2002; Irizarry *et al.*, 2003; Law *et al.*, 2014; Love *et al.*, 2014; Risso *et al.*, 2014; Robinson and Oshlack, 2010; Wagner *et al.*, 2012).

In particular, the voom method (Law *et al.*, 2014) stabilizes variance of RNA-seq data. It does so by identifying the mean–variance relationship of the data at the gene-level. It fits a gene-wise linear model to calculate the residual standard deviation. Then, it fits a LOWESS (locally weighted regression) to each residual standard deviation, which is a function of the average normalized values for each gene to extract the mean–variance relation. Finally, it interpolates the mentioned trend for predicting all normalized log-cpm values’ variances. However, voom does not apply to genomic signals such as ChIP-seq and ATAC-seq. Unlike voom which stabilizes the variance of the data at the gene-level, VSS aims to do so at the whole-genome level. In particular, the LOWESS-based curve fitting procedure used by voom does not scale to genome-scale data, so VSS uses a weighted average followed by spline fit (Section 2).

## 2 Materials and methods

### 2.1 ChIP-seq data

We acquired ChIP-seq data from the ENCODE consortium (encodeproject.org, Supplementary Section A) for the histone modification H3K4me3 on 11 cell lines: GM12878, H1-hESC, HUVEC, K562, NHLF, GM06990, HCPEpiC, AG09319, NHEK, HMEC and HSMM. We also used histone modifications H3K36me3, H3K4me1, H3K27me3 and H3K9me3 on H1-hESC cell line. Histone modification H2AFZ was used on cell lines NHEK and HSMM. In addition, we used histone modification H3K79me2 on NHEK, HSMM and HMEC cell lines. We also used histone modification H3K9me3 on four cell lines: NHEK, AG04450, HMEC and HSMM. Finally, H3K36me3 histone modification was used on HMEC cell line. ENCODE accession number of these assays is provided in the Supplementary Table S1. These ChIP-seq datasets were processed with a uniform pipeline (ENCODE Project Consortium, 2012). Briefly, the ChIP-seq reads were mapped to the hg19 reference genome and reads were shifted and extended according to the estimated fragment length to produce a read count for each genomic position. As controls, ChIP-seq input experiments were performed by the same labs. Two signals were produced: FE and log p-value. FE signal is defined as the ratio of observed data over control (Hoffman *et al.*, 2012b). *P*-value signal is defined as the log p-value of a Poisson model with a null distribution derived from the control (Kundaje *et al.*, 2015).

### 2.2 RNA-seq data

For use in evaluation, we acquired RNA-seq datasets for each of the cell types above from the Roadmap Epigenomics consortium (Kundaje *et al.*, 2015). These RNA-seq datasets were processed with a uniform pipeline that produces a TPM (Transcripts Per Million) value for each gene (Kundaje *et al.*, 2015). To stabilize the variance of these signals, we used an asinh transformation.

### 2.3 Identifying the mean–variance relationship

**Fig. 1.**
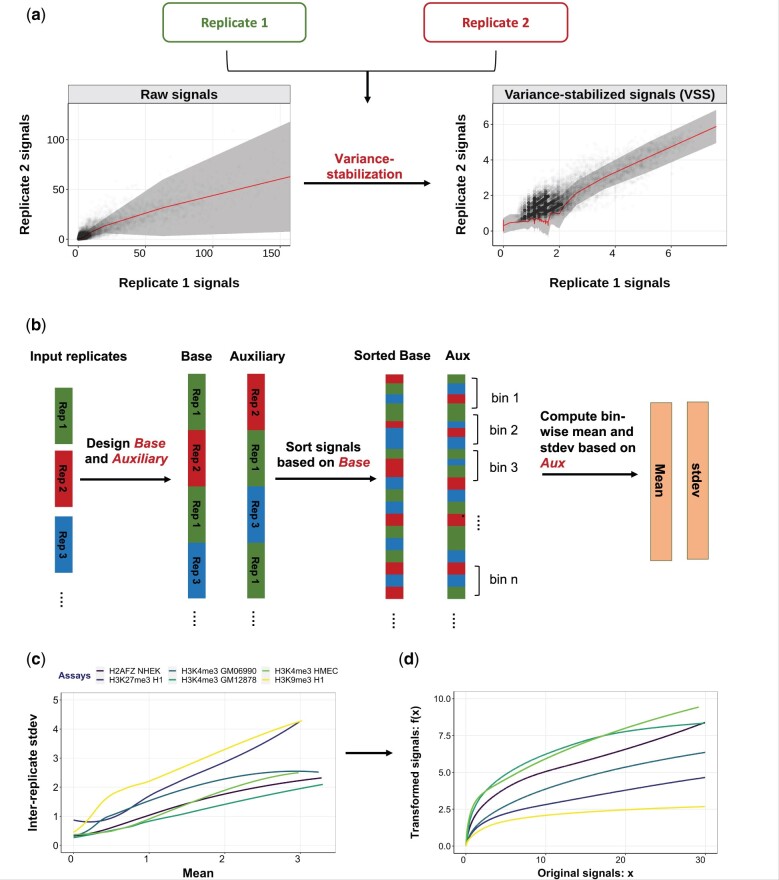
General schematic of the VSS method. (**a**) Replicate 1 versus replicate 2 signals in H3K4me3 HSMM before and after VSS transformation. Shaded area represents the average variance in replicate 2 for a given value of replicate 1; variation in width of shaded region indicates a nonuniform mean–variance relationship. (**b**) VSS uses two or more replicates to learn the empirical mean–variance relationship of the input data. For *M* replicates, VSS defines *base* and *auxiliary* vectors according to all M(M−1) possible combinations of the replicates. Then, it identifies the mean–variance relationship by computing the bin-wise mean and standard deviation from *auxiliary* vector. (**c**) Learned mean–variance relationships for several datasets. Horizontal and vertical axes denote mean and standard deviation, respectively. Note that the mean–variance relationship differs across datasets, indicating that each requires a different transformation. (**d**) Learned transformation functions. Horizontal and vertical axes indicate input and output values, respectively

Our variance-stabilizing transformation depends on determining the mean–variance relationship for the input dataset. We learn this relationship by using multiple replicates of the same experiment. We define two vectors, $x^{\left(\text{base}\right)}$ and $x^{\left(\text{aux}\right)}$ that capture replicated signals. Specifically, for each distinct pair of replicates $x^{\left(i\right)},x^{\left(j\right)}$ where i≠j, we concatenate $x^{\left(i\right)}$ to $x^{\left(\text{base}\right)}$ and $x^{\left(j\right)}$ to $x^{\left(\text{aux}\right)}$. Thus, $x^{\left(\text{base}\right)}$ and $x^{\left(\text{aux}\right)}$ are each vectors of length NM(M−1), for *M* replicates and a genome of length *N*. Base-aux pairs $\left(x_{i}^{\left(\text{base}\right)},x_{i}^{\left(\text{aux}\right)}\right)$ represent every possible pair of replicated signals (Fig. 1b).

Let the observed signal at position *i* be $x_{i}^{\left(\text{base}\right)}$ and $x_{i}^{\left(\text{aux}\right)}$ for the base and auxiliary, respectively. Our model imagines that every position *i* has an unknown distribution of sequencing reads for the given assay *x_i_*, which has mean $\mu_{i}=\text{mean}(x_{i})$. We further suppose that there is a relationship σ(μ) between the mean and variance of these distributions. That is, $\text{var}(x_{i})=\sigma {\left(\mu_{i}\right)}^{2}$. We are interested in learning σ(μ). Observe that *x_i_* is an unbiased estimate of *μ_i_*, and that ${\left(x_{i}^{\left(\text{base}\right)}-x_{i}^{\left(\text{aux}\right)}\right)}^{2}$ is an unbiased estimate of $\sigma {\left(\mu_{i}\right)}^{2}$. We use this observation to estimate the function σ(μ) as follows.

We first sort the NM(M−1) genomic signals i∈{1…NM(M−1)} by the value of $x_{i}^{\left(\text{base}\right)}$ and define bins with *b* genomic signals each.

Let $I_{j}\subseteq \{1\ldots NM(M-1)\}$ be the set of positions in bin *j*. For each bin *j*, we compute $\mu_{j}=1/b\sum_{i\in I_{j}}x_{i}^{\left(\text{aux}\right)}$ and $\sigma_{j}^{2}=1/b\sum_{i\in I_{j}}{\left(x_{i}^{\left(\text{aux}\right)}-\mu_{j}\right)}^{2}$. To increase the robustness of these estimates, we smooth across bins by defining

(1) $${\bar{\sigma }}_{j}^{2}=\frac{\sum_{i=j-w}^{j+w}2^{-b|j-w|/\beta }\sigma_{i}^{2}}{\sum_{i=j-w}^{j+w}2^{-b|j-w|/\beta }}.$$

That is, we take the weighted average of 2w+1 bins centered on *j*, where bin *j* + *k* has weight $2^{-bk/\beta }$. *β* is a bandwidth parameter—a high value of *β* means that weight is spread over many bins, whereas a low value means that weight in concentrated on a small number of bins. We define the window size *w* such that it includes bins with weight at least 0.01; specifically, w=−β log(0.01)/b log(2).

The choice of *b* and *β* forms a bias-variance trade-off. Larger values of *b* and *β* lead to more observations contributing to each estimate ${\bar{\sigma }}_{j}$ and therefore result in a lower variance. In contrast, small values of *b* and *β* lead to a very homogeneous set of positions *I_j_* and therefore less averaging across dissimilar positions.

Most genomic signals are zero-inflated. That is, a large fraction of positions have zero signal. To account for this pattern, we defined a separate bin for zero-signal positions $I_{0}=\{i|x_{i}^{\left(\text{base}\right)}=0\}$ and defined *σ*_0_ and *μ*_0_ as above. We used this zero bin for raw and FE signals, but not log Poisson p-value (LPPV), which are not zero-inflated.

We used a smoothing spline to fit an estimated mean–variance curve $\hat{\sigma }(x)$. A smoothing spline estimator implements a regularized regression over the natural spline basis. We fit a function $\hat{\sigma }(\mu )$ using the estimated values of ${\bar{\sigma }}_{j}$. The spline coefficients *w* are selected to

$$\text{minimize}\;(1-p)\sum_{j}w_{j}{\left({\bar{\sigma }}_{j}-\hat{\sigma }(\mu_{j})\right)}^{2}+p\int {\left(\frac{d^{2}\hat{\sigma }(\mu )}{dx^{2}}\right)}^{2}dx,$$

where *μ* and $\bar{\sigma }$ are a set of observations obtained from mean–variance data points. Variables $\hat{\sigma }(\mu )$, *w* and *p* represent smooth spline curve, weight coefficients and smoothing parameter, respectively. The variable *p* parameter varies between (0,1] such that *p* = 0 results in a cubic spline with no smoothing, and when *p* approaches zero the result is a linear function.

To find the optimum value of *spar* parameter (*p*), first the smooth.spline function is called by activating the cross-validation in the smooth.spline (CV = TRUE). Following the cross-validation procedure, *spar* parameter is returned as the smoothing factor. We identified the optimal curve using the R function call smooth.spline(means, sigmas, spar = *p*).

We examined a number of methods for identifying the mean–variance relation from multiple replicates. We discussed the details of the examined approaches in the Supplementary Section E.

We performed a hyperparameter search to choose *b* and *β* (Supplementary Figs S1 and S2), using the log likelihood and variance-instability metrics (defined below). We chose $\beta =10^{3}$ and $b=10^{5}$ for zero-inflated signals (raw and FE) and $\beta =10^{7}$ and $b=10^{3}$ for nonzero-inflated signals (LPPV). Note that in all of the evaluations, VSS models are trained on chromosome 22 and tested on chromosome 21.

### 2.4 Calculating variance-stabilized signals

Having learned the mean–variance relationship, we compute VSS using the variance-stabilizing transformation (Durbin *et al.*, 2002)

(2) $$t(x)=\int_{0}^{x}\frac{1}{\hat{\sigma }(u)}du,$$

where *x* is an untransformed signal and $\hat{\sigma }(u)$ is the learned standard deviation for a signal with mean *u*. This transformation is guaranteed to be variance-stabilizing; i.e. $\text{var}(t(x_{i}))$ is constant for all genomic positions *i*.

### 2.5 Alternative transformations

To attempt to stabilize the variance, existing methods usually apply either a log or arcsinh transformation. These transformations are used because they are variance-stabilizing for certain mean–variance relationships (Bartlett, 1947). Specifically, log(x) is variance-stabilizing when σ(μ)=sμ for some constant *s*, and arcsinh(x) is variance-stabilizing when $\sigma (\mu )=s\sqrt{\mu^{2}+1}$ (Box, 1953). In the experiments below, we compare to both existing units under both existing transformations. We evaluated the performance log with a general linear transformation (log(ax+b)). We found that doing so did not improve results (Supplementary Section C), so we focused on the standard offset log(x+1).

### 2.6 Variance quality-of-fit evaluation

A transformation implicitly assumes that a dataset has a specific mean–variance relationship. The assumed variance $\tilde{\sigma }(u)$ for a given value *u* equals the inverse of the derivative of the transformation (Section 2.4)

(3) $${\tilde{\sigma }}_{t}(u)=\frac{1}{\frac{d}{du}t(u)}.$$

As noted above, a log(*x* + 1) transformation implicitly assumes the mean–variance relationship $\tilde{\sigma }(\mu )=\mu +1$ and the arcsinh(*x*) transformation assumes the mean–variance relationship $\tilde{\sigma }(\mu )=\sqrt{\mu^{2}+1}$.

To measure the quality-of-fit of an assumed mean–variance relationship, we evaluated the data log likelihood under the assumed $\tilde{\sigma }$.

Specifically, the log likelihood of a given dataset is defined as

(4) $$\sum_{i}\;\text{log}\;N(x_{i}^{\left(\text{aux}\right)}|\mu =x_{i}^{\left(\text{base}\right)},\sigma =\hat{\sigma }(x_{i}^{\left(\text{base}\right)})).$$

A Gaussian distribution appears in this expression because that is the max-entropy distribution with a specific mean and variance. This value is maximized when the inferred variance equals the variance of the data.

### 2.7 Variance-instability evaluation

To evaluate whether a given transformation achieves a uniform mean–variance relationship, we defined the following variance-instability metric. Let $t(x_{i}^{\left(\text{base}\right)})$ and $t(x_{i}^{\left(\text{aux}\right)})$ be the transformed signals at the *i*th genomic position. Using the binning approach described above, we divided genomic positions to *B* bins of increasing value of $t(x_{i}^{\left(\text{base}\right)})$, where each bin is of size *b* = 10 000.

Let *v_j_* be the mean squared difference between replicates for positions in bin *j*,

(5) $$v_{j}=\sum_{i\in \text{bin}\;j}{\left(t(x_{i}^{\left(\text{base}\right)})-t(x_{i}^{\left(\text{aux}\right)})\right)}^{2}.$$

Let *σ*_1_ and *σ*_2_ be the standard deviation of $t(x^{\left(\text{base}\right)})$ and $t(x^{\left(\text{aux}\right)})$, respectively. We define the variance-instability metric as the scaled variance of *v_j_* across bins,

$$\text{variance}-\text{instability}(t)=\frac{1}{\sigma_{1}^{2}\sigma_{2}^{2}}\text{var}(v_{1:B}).$$

The $\frac{1}{\sigma_{1}^{2}\sigma_{2}^{2}}$ factor normalizes for the variance of the transformed signal; without this factor, *t*(*x*) and αt(x) (for a constant *α*) have different variance instability. Signals with unstable variance will have large values of the variance-instability metric.

### 2.8 Segmentation and genome annotation (SAGA) evaluation

As described above, SAGA algorithms are sensitive to the mean–variance relationship in the input datasets. SAGA algorithms take as input a collection of signals for a given biosample. They partition the genome and assign a label to each segment such that positions with the same label have similar patterns in the input datasets. SAGA algorithms are widely used to integrate datasets and annotate regulatory elements.

To evaluate the quality of annotations produced by signals under a given transformation, we defined the following SAGA metric. Following previous work (Zhang and Hardison, 2017), we quantified quality of an SAGA annotation according to the strength of the relationship between the annotation of a genic region with that gene’s expression.

Specifically, for a collection of signals from a given biosample, we used the SAGA algorithm Segway (Hoffman *et al.*, 2012a) to produce an annotation. This annotation assigns one of *k* integer labels $l_{i}\in \{1..k\}$ to each genomic position *i*. We defined features for each gene as follows. We divided each genic region into 20 bins by dividing the transcribed region into 10 equally spaced bins and defining five 1 kb bins upstream of the transcription start site (TSS) and downstream of the transcription termination site (TTS), respectively. We defined features $f_{b,k}$ for each bin *b* as a one-hot encoding of the majority label in each bin. That is, this process associates each gene with a vector of 20*k* features.

We trained an Extreme Gradient Boosting (XGBoost) regression model to predict a gene’s RNA-seq expression (Section 2.8) value from this vector of features. We trained a regression model on a matrix containing all genes in a chromosome. As features, we used a one-hot encoding feature vector that indicates 1 in the corresponding position of the predicted label and 0 elsewhere. For each bin, we considered the majority feature vector as a representation of that bin’s annotation. We used the coefficient of determination (*r*^2^) to quantify the predictive power of this regressor.

For each transformation method, we used four different values k={3,5,10,15} for number of the labels to be predicted by annotation. We considered four biosamples: H1-hESC, NHEK, HSMM and HMEC. We used all available replicated histone modification data for each biosample: We used H3K36me3, H3K4me3, H3K9me3, H3K27me3 and H3K4me1 for H1-hESC. We used H2AFZ, H3K4me3, H3K9me3 and H3K79me2 for NHEK. We used H2AFZ, H3K4me3, H3K9me3 and H3K79me2 for HSMM. We used H3K36me3, H3K4me3, H3K9me3 and H3K79me2 for HMEC.

## 3 Results

### 3.1 Genomic signals are not variance-stabilized

To evaluate whether existing units for genomic signals have stable variance, we computed the mean–variance relationship for a number of existing datasets (Fig. 1c). As we expected, we found that the variance has a strong dependence on the mean; genomic positions with low signals experience little variance across replicates, whereas positions with high signals experience much larger variance (Fig. 1c). Moreover, the relationship does not match that expected by the currently used log(x+1) and asinh(x) transformations. For example, the former transformation assumes a linear relationship (Section 2). The observed mean–variance relationship does not precisely match the relationships assumed by either transformation, indicating that neither of these transformations is fully variance-stabilizing (Fig. 1c).

**Fig. 2.**
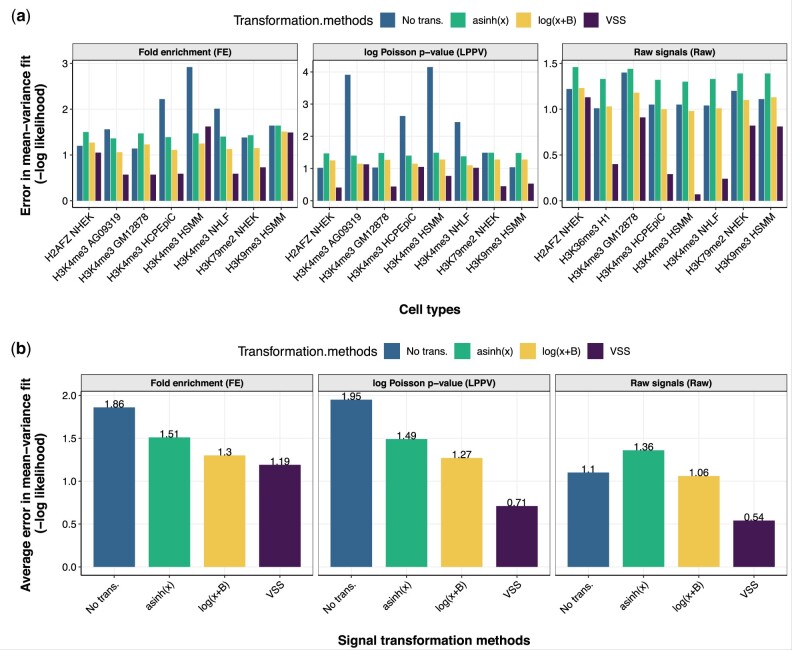
(**a**) Goodness of fit to the mean–variance relationship derived from FE signals, LPPV and raw signals (Raw), measured by Gaussian log likelihood (Section 2). Lower values of negative log likelihood indicate better fit. Log likelihood was computed on chromosome 21; VSS’s mean–variance relationship was trained on chromosome 22. (**b**) Same as (a), but averaged across datasets for FE signals, LPPV and raw signals (Raw)

The observation that existing transformations are not variance-stabilizing was confirmed when we quantified this fit (Fig. 2). To measure the accuracy of a variance estimate, we used the log likelihood of a given mean–variance relationship estimate, which is maximized when the inferred variance equals the variance of the data (Section 2). As expected, we found that a uniform variance model implied by using untransformed signals had a poor likelihood (average log density of –1.9), reflecting nonuniform variance (Fig. 2b, panel FE). We found that the variance estimates from the log(x+1) and asinh(x), where *x* is the FE signal, greatly improved the likelihood (average log density of –1.3 and –1.5, respectively). However, we found that mean–variance relationship learned by VSS had much better likelihood (average log density –1.2) than either transformation, indicating that the learned curve successfully models the mean–variance relationship of the data (Fig. 2b, panel FE). We found that VSS’s mean–variance fit was also better than log or asinh when using either raw reads or LPPV as the base units (Fig. 2b).

Moreover, we found that the mean–variance relationship differs greatly between experiments. For many histone modification ChIP-seq experiments like H3K4me3 in HSMM, a log transformation yields nearly optimal fit, indicating that the data have a nearly linear mean–variance relationship (Fig. 2a, panel FE). However, other experiments like H3K4me3 in GM12878 and H2AFZ in NHEK, have a very nonlinear mean–variance relationship (Fig. 2a, panel FE). In fact, for some experiments, a log or asinh transformation has worse fit than no transformation, indicating that these transformations actually destabilize the variance (Fig. 2a, panel FE). Future work should investigate what properties of an experiment determine its mean–variance relationship. The mean–variance relationship learned by VSS correctly captures these differences, as indicated by its good likelihood on all datasets. These differences indicate that it is necessary to learn a separate mean–variance relationship for each dataset, rather than applying a single transformation (such as log or asinh) to every dataset.

### 3.2 Differences between replicates are stabilized after transformation

**Fig. 3.**
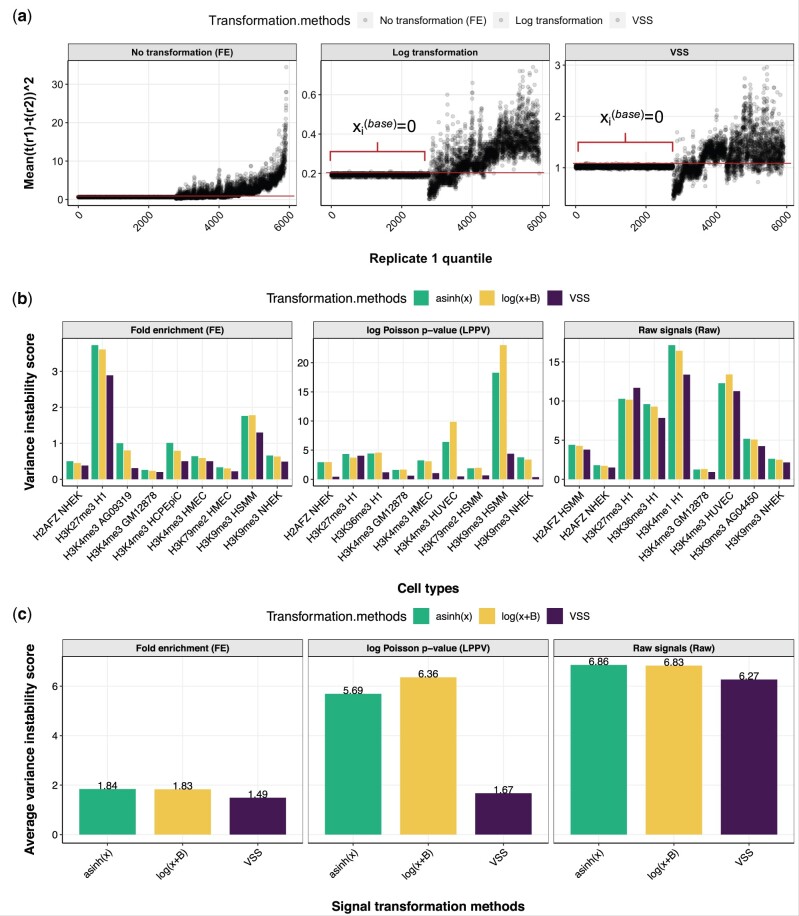
Variance instability of transformed signals. (**a**) Each point corresponds to a bin, where binning is defined according to replicate 1 value (Section 2). Horizontal axis indicates binning index. Vertical axis indicates squared difference of values between replicates in H3K9me3 NHEK (SSD (Sum of squared differences) depends on the scale of the data). The flat line on the left half of each plot corresponds to positions where $x^{\left(\text{base}\right)}=0$. Signals with stable variance show a flat (constant) trend on this plot; a trend (increasing or decreasing) indicates unstable variance. (**b**) Variance-instability score on FE signals, LPPV and raw signals (Raw) (Section 2). Lower values indicate more stable variance. (**c**) Same as (b), but averaged across experiments on FE signals, LPPV and raw signals (Raw). VSS’s mean–variance relationship was trained on chromosome 22 and variance-instability score was computed on chromosome 21. We omitted the variance-instability value for untransformed signals because its value would distort the vertical axis (mean of 601, 117 and 1877 across experiments for FE signals, LPPV and raw signals, respectively)

To measure whether a given transformation stabilizes variance in a given signal dataset, we defined the variance-instability metric (Section 2.7). This metric measures the degree to which differences between replicates vary for different magnitude of signal. In other words, this metric quantifies the consistency of the variance of mean squared between-replicate differences, among bins which divide the signal values in equally spaced groups. A lower value of the variance-instability metric indicates that the transformation has been successful in stabilizing the variance of the dataset. We found that signals transformed using VSS have better (lower variance-instability score) variance stability than either untransformed signals or signals after alternative transformations (Fig. 3).

FE signals transformed by either log(*x* + 1) and asinh(*x*) had an average of 1.8 variance instability, whereas VSS have instability of 1.5 (Fig. 3c, panel FE).

Changing the offset of the log transformation—log(ax+b)—did not substantially improve results for any choice of *a* or *b* (Supplementary Figs S3 and S4). This indicates that VSS units have more consistent signals among different replicates of an experiment (Fig. 3b and c). This pattern also holds when using Raw or LPPV as the base signal (Fig. 3, panels LPPV and Raw).

To investigate the sensitivity of VSS’s results to experiment quality, we evaluated its results on experiments with varying quality according to ENCODE’s quality scores (Supplementary Section F). We found that results were similar across different quality scores (Supplementary Fig. S8).

### 3.3 VSS improve SAGA algorithms

To evaluate the efficacy of transformed signals as input to Gaussian models, we use SAGA as an example. SAGA algorithms are widely used to integrate genomic datasets and annotate genomic regulatory elements (Chan *et al.*, 2018; Hoffman *et al.*, 2012a, 2012b; Zhang and Hardison, 2017; Zhang *et al.*, 2016). Following previous work (Libbrecht *et al.*, 2019; Zhang *et al.*, 2016), we evaluated the quality of an annotation by the correlation between the label of a gene body and whether that gene is expressed as measured by RNA-seq (Section 2). We evaluated this correlation across multiple cell types and model initializations (Section 2). We believe that high-quality input signals will lead to a high-quality annotation.

We used the SAGA algorithm Segway (Hoffman *et al.*, 2012a) annotation for this analysis.

**Fig. 4.**
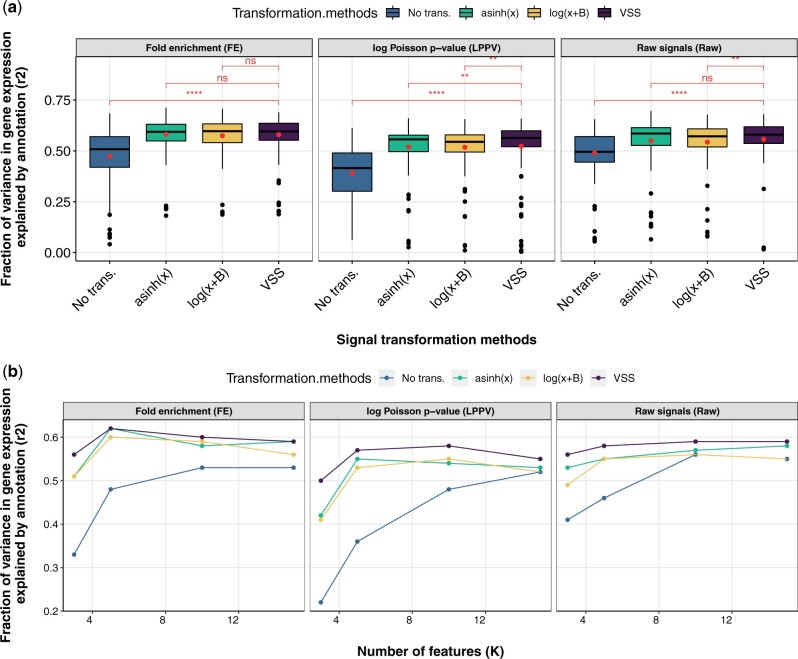
Evaluation of annotations relative to gene expression. Vertical axis is the fraction of variance in gene expression explained (*r*^2^, genome annotation evaluation section). (**a**) Horizontal axis is the transformation methods on FE signals, LPPV and raw signals (Raw). Brackets indicate significance of VSS transformation to the other methods according to p-value from paired one-sided Wilcoxon signed rank test. (**b**) Horizontal axis is the number of features or states in a given model, respectively (k). Box plots for representing the variance of the points can be found in Supplementary Figure S11. Results are shown on chromosome 21 for an VSS model trained on chromosome 22

We found that using variance-stabilized signals from VSS improves annotations produced by SAGA algorithms (Fig. 4). As had been previously observed (Hoffman *et al.*, 2012a), using nonstabilized FE signal results in poor performance (mean *r*^2^ = 0.47, Fig. 4). To account for this, Segway recommends using an asinh transform; doing so substantially improves performance (mean *r*^2^ = 0.57, Fig. 4). VSS produces similar results to asinh on FE data (mean *r*^2^ = 0.57, *p* = 0.28). However, VSS outperforms asinh when using LPPV as the base signals (*p* = 0.0064, paired one-sided Wilcoxon signed rank test). Likewise, VSS outperforms a log transformation for LPPV and raw signals (*p* = 0.0031 and *p* = 0.0009, respectively). This improvement likely results from the fact that VSS stabilizes variance in all cases, whereas asinh does so only when datasets happen to have a specific mean–variance relationship.

## 4 Discussion

In this manuscript, we proposed VSS, a method that produces units for sequencing-based genomic signals that have the desirable property of variance stability. We found that the transformations that are currently used to stabilize variance—log(x+1) and asinh(x)—do not fully do so. In fact, we found that the mean–variance relationship of genomic signals varies greatly between datasets, indicating that no single transformation can be applied to all datasets uniformly. Instead, variance stability requires a method such as VSS that empirically determines the experiment-specific mean–variance relationship.

We showed that VSS successfully stabilizes variance in genomic datasets. Further, we found that using variance-stabilized data improves the performance of Gaussian models such as SAGA.

Variance-stabilized signals will aid in all downstream applications of genomic signals. In particular, they are valuable for three reasons. First, VSS allow downstream methods to use MSE loss or Gaussian likelihood distributions, which are much easier to optimize than the existing practice of implementing a model that accounts for the mean–variance relationship. This will improve tasks that currently use Gaussian models, such as chromatin state annotation and imputation.

Second, VSS can be easily analyzed by eye because the viewer does not need to take the mean–variance relationship into account when visually inspecting the data. For example, when viewing genomic signals in a genome browser, variance-unstable signals often exhibit high peaks that swamp the vertical axis and flatten other variations in signal (Supplementary Fig. S5). Existing methods for handling this problem—using a log/asinh transform or cutting off the vertical axis—can also be effective, but they lack the principled basis of VSS.

Third, VSS overcomes the problems that other signal transformation methods like log transformation may cause. When transforming genomic signals, a trade-off must be considered. One must (i) reduce outlier spikes in signals so they do not dominate analysis while (ii) avoiding overly amplifying background noise. VSS provides a principled way to make this trade-off; it reduces signals by exactly the amount needed to stabilize variance.

A key limitation of VSS is that it requires the availability of replicated data. A fruitful direction for future work might aim to remove this dependence, e.g. by training a consensus transformation to apply across non-replicated data. Another direction for resolving this issue may be to use the autocorrelation in the genome, as neighboring positions in a replicate, can be considered as pseudo-replicates of one another. Doing so would eliminate the need for multiple replicates for identifying the mean–variance relationship, as this trend can be identified from a single replicate.

A related limitation is that VSS relies heavily on the comparability of its input replicates. For example, if a pair of replicates exhibits completely irreproducible peaks or there are (e.g.) batch effects between the replicates, VSS will estimate extremely high variance overall, leading to very low-magnitude signals.

Moreover, it would be beneficial to investigate if VSS can stabilize the variance between different experiments. This task would be specifically useful in the downstream analyses in which selecting a region as significant depends on the between-replicates-variance. Future work may focus on figuring out if one can use the same mean–variance relationship trained on one experiment to get the VSS for different samples.

Another related issue concerns standardizing dynamic range of different experiments. VSS has not been designed to address this issue itself. However, a user can use VSS as part of a pipeline with cross-experiment normalization methods such as S3norm (Xiang *et al.*, 2020) to stabilize both variance and standardize dynamic range.

## Funding

This was supported by a Simon Fraser University President’s Research Grant and a NSERC CREATE scholarship. 

*Conflict of Interest*: none declared.

## Supplementary Material

btab457_Supplementary_Data
