# Supplementary material for: VSS: variance-stabilized signals for sequencing-based genomic signals
Source: Bioinformatics. 2021 Jun 24;37(23):4383–91. doi: 10.1093/bioinformatics/btab457 (PMC8652025; doi:10.1093/bioinformatics/btab457)
Supplement: btab457_Supplementary_Data [file btab457_supplementary_data.pdf]

Supplementary Information

A Datasets Information

Table 1. Genomic assays ENCODE accession numbers.

Assay/Celltype	ENCODE accession number
H3K4me3 GM1287	ENCSR000AKA
H3K4me3 H1-hESC	ENCSR000AMG
H3K4me3 HUVEC	ENCSR000AKN
H3K4me3 K562	ENCSR000AKU
H3K4me3 NHLF	ENCSR000DWZ
H3K4me3 GM06990	ENCSR000DQV
H3K4me3 HCPEpiC	ENCSR000DTN
H3K4me3 AG09319	ENCSR000DPU
H3K4me3 NHEK	ENCSR000ALO
H3K4me3 HMEC	ENCSR016JWS
H3K4me3 HSMM	ENCSR000ANK
H3K36me3 H1-hESC	ENCSR925LJZ
H3K4me1 H1-hESC	ENCSR631RJR
H3K27me3 H1-hESC	ENCSR216OGD
H3K9me3 H1-hESC	ENCSR883AQJ
H2AFZ NHEK	ENCSR000ARL
H2AFZ HSMM	ENCSR000APA
H3K79me2 NHEK	ENCSR000ARM
H3K79me2 HSMM	ENCSR000ANQ
H3K79me2 HMEC	ENCSR000ASB
H3K9me3 NHEK	ENCSR000ARN
H3K9me3 AG04450	ENCSR000DPJ
H3K9me3 HMEC	ENCSR000ARG
H3K9me3 HSMM	ENCSR000ANR
H3K36me3 HMEC	ENCSR000ALY

B Setting VSS hyperparameters

In order to identify optimum values for VSS's hyperparameters, we evaluated many possible combinations of values using the two evaluation measures, likelihood analysis and variance instability (Figure 1). The set of parameters is considered optimized if they minimize the two evaluation metrics. Thus, based on these results, we chose $\beta = 10^3$ and $b = 10^5$ as the optimal set of the parameters as it satisfies all evaluation metrics simultaneously. We have also compared the optimization results with $\log(x+1)$ transformation which is shown by red lines in the Figure 1. The results indicate that our approach is outperforming the $\log(x+1)$ transformation in all investigated evaluation metrics. The parameter setting results of the first mode is shown in Figure 1. Based on the experiments, we chose to use $\beta = 10^3$ and $b = 10^5$ as the best combination of the parameters for the first mode since it optimizes all evaluation metrics simultaneously.

For the second mode of the mean-variance relationship identification, we considered all the genomic signals in the smoothing procedure rather than considering the fluctuated signals in one bin. Thus, based on two criteria, we chose $\beta = 10^7$ and $b = 10^3$ as the optimal set of the parameters for the second mode as it satisfies all evaluation metrics simultaneously. We have also compared the optimization results with $\log(x+1)$ transformation which is shown by red lines in the Figure 2.

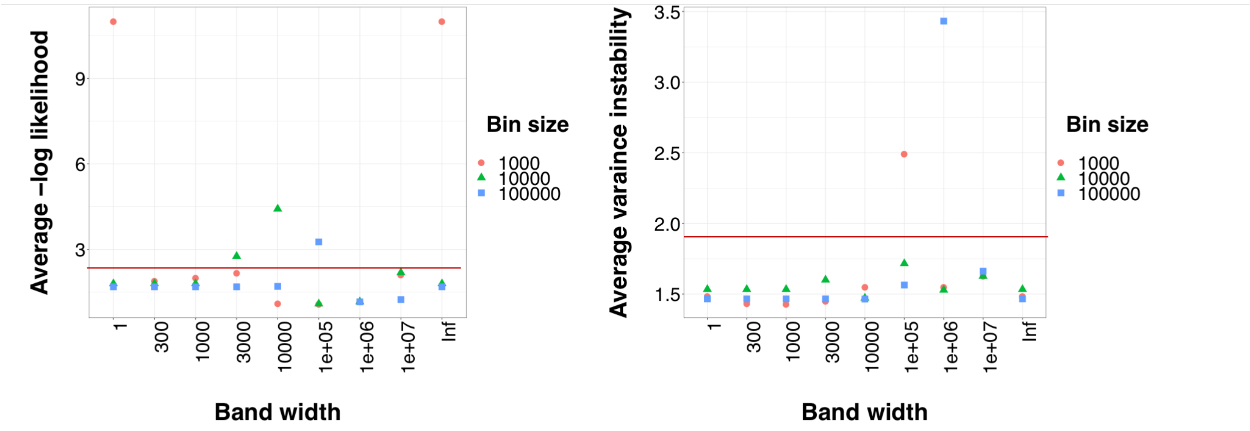

Fig. 1. Relationship between bin size and bandwidth in (a) likelihood analysis (b) variance instability analysis in zero-inflated signals. Among the band width values, the value Inf indicates that we applied no smoothing on the curve (Unweighted mean-variance curve). Red line indicates the performance of $\log(x+1)$ transformation.

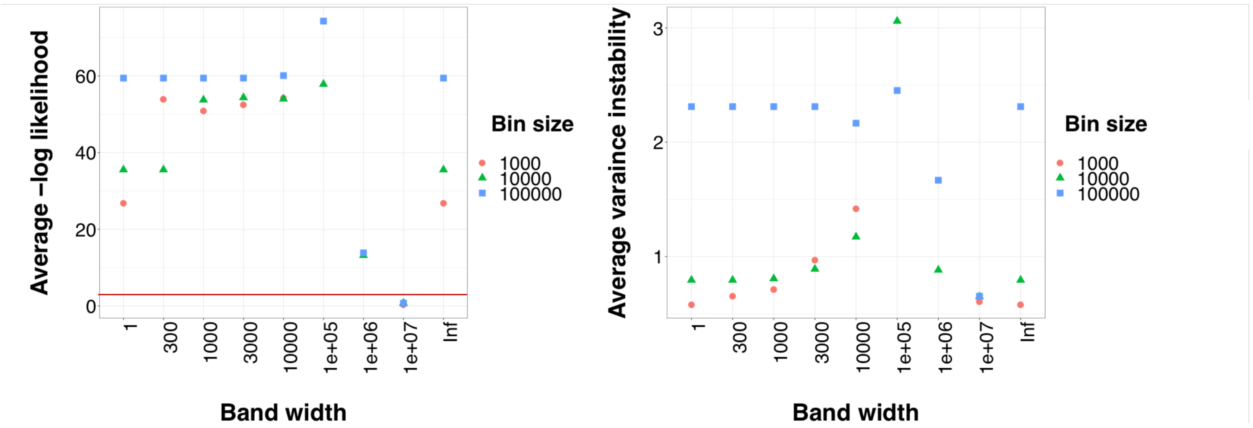

Fig. 2. Relationship between bin size and bandwidth in (a) likelihood analysis (b) variance instability analysis in signals that are not zero-inflated. Among the band width values, the value Inf indicates that we applied no smoothing on the curve (Unweighted mean-variance curve). Red line indicates the performance of $\log(x+1)$ transformation. Average variance instability score for $\log(x+1)$ is 6.4 .

C Alternative offsets for a log transformation, $\log(ax + b)$

We evaluated whether it is possible to improve the log transform using a linear transform $\log(ax + b)$. We did so using the previously-described log likelihood and variance instability evaluations. We found that no single set of parameters performed best across all data sets (Supplementary Figures 3, 4), and all performed less well than VSS.

we could not identify a single pair of parameters that can optimize both criteria. Therefore, we believe that default choice of $a = 1$ and $b = 1$ can be applied to the $\log(ax + b)$

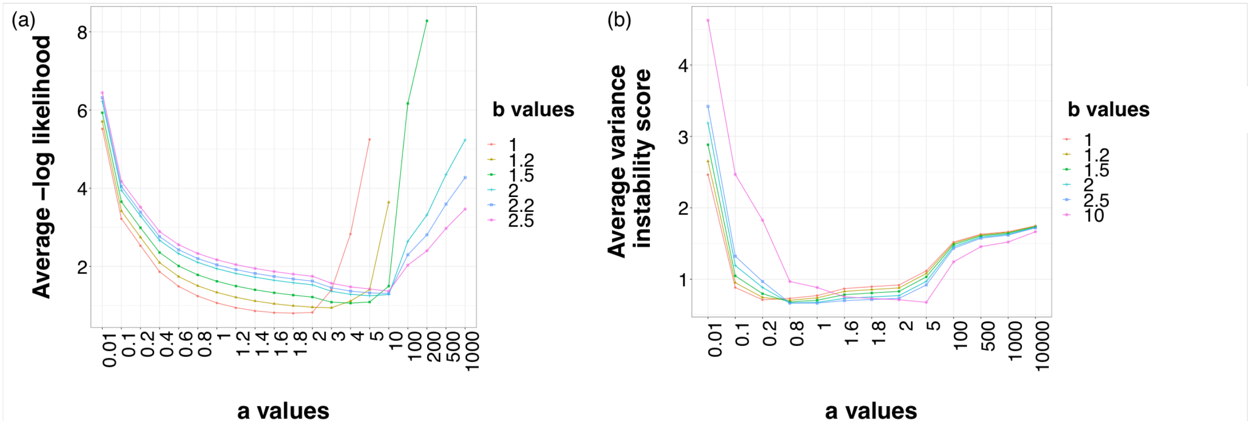

Fig. 3. Relationship between a and b in $\log(ax + b)$ transformation in (a) likelihood analysis (b) variance instability analysis .

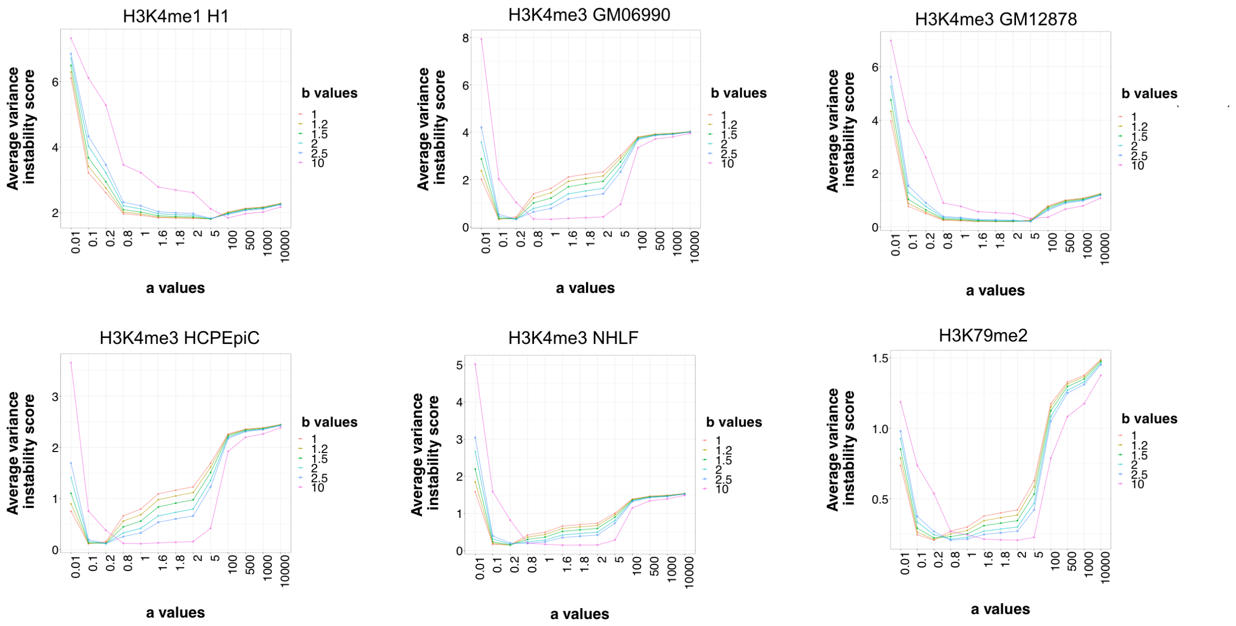

Fig. 4. Relationship between a and b in $\log(ax + b)$ transformation in variance instability analysis .

D Visualization of UCSC tracks

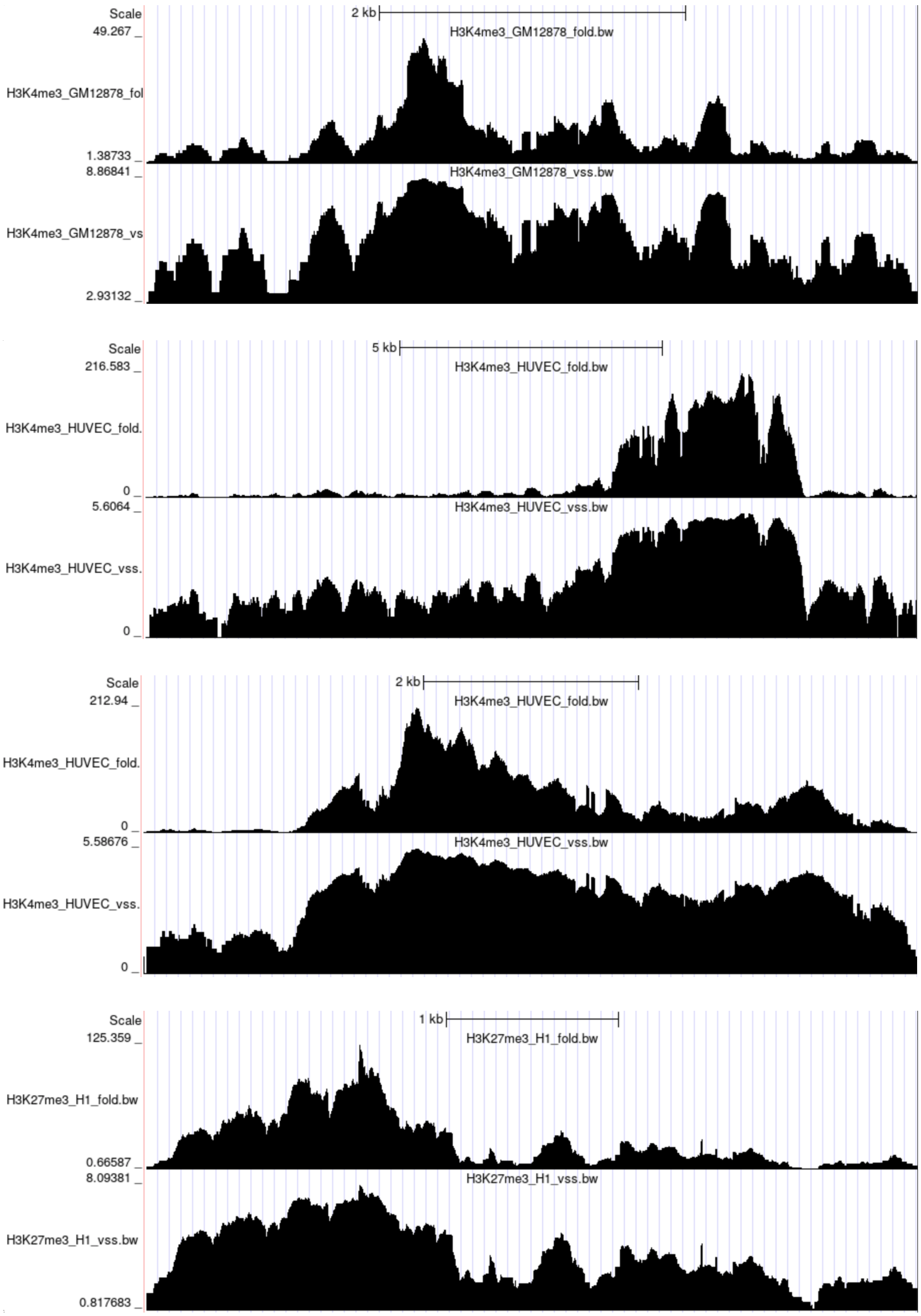

Fig. 5. Visualization of genomic signals in the UCSC genomic browser.

E Alternative variations of VSS

We evaluated multiple variations of VSS to identify the best fit for the mean-variance relation of the data. We did so by comparing the average performance of the proposed methods in log likelihood and variance instability evaluations (Figure 6). As mentioned before (Methods), *base* and *auxiliary* are derived by concatenating every possible combination of all M replicates available.

One of the alternative variations of VSS is to use *base* for sorting the genomic signals and calculate the mean and variance of the data using the average signals of *base* and *auxiliary* (Figure 6, VSS (Mean of Base and Aux)). The other variation of VSS is not to introduce *base* and *auxiliary* but to use replicate 1 and replicate 2 signals. By using this approach, signals are sorted based on replicate 1 values while mean and variance of the data are computed using the average signals of replicate 1 and 2 (Figure 6, VSS (Mean of Rep1 and Rep2)). Based on both criteria (log likelihood and variance instability evaluations), the approach introduced in the manuscript (VSS) has the best performance among all variations and captures the best fit for the mean-variance relationship.

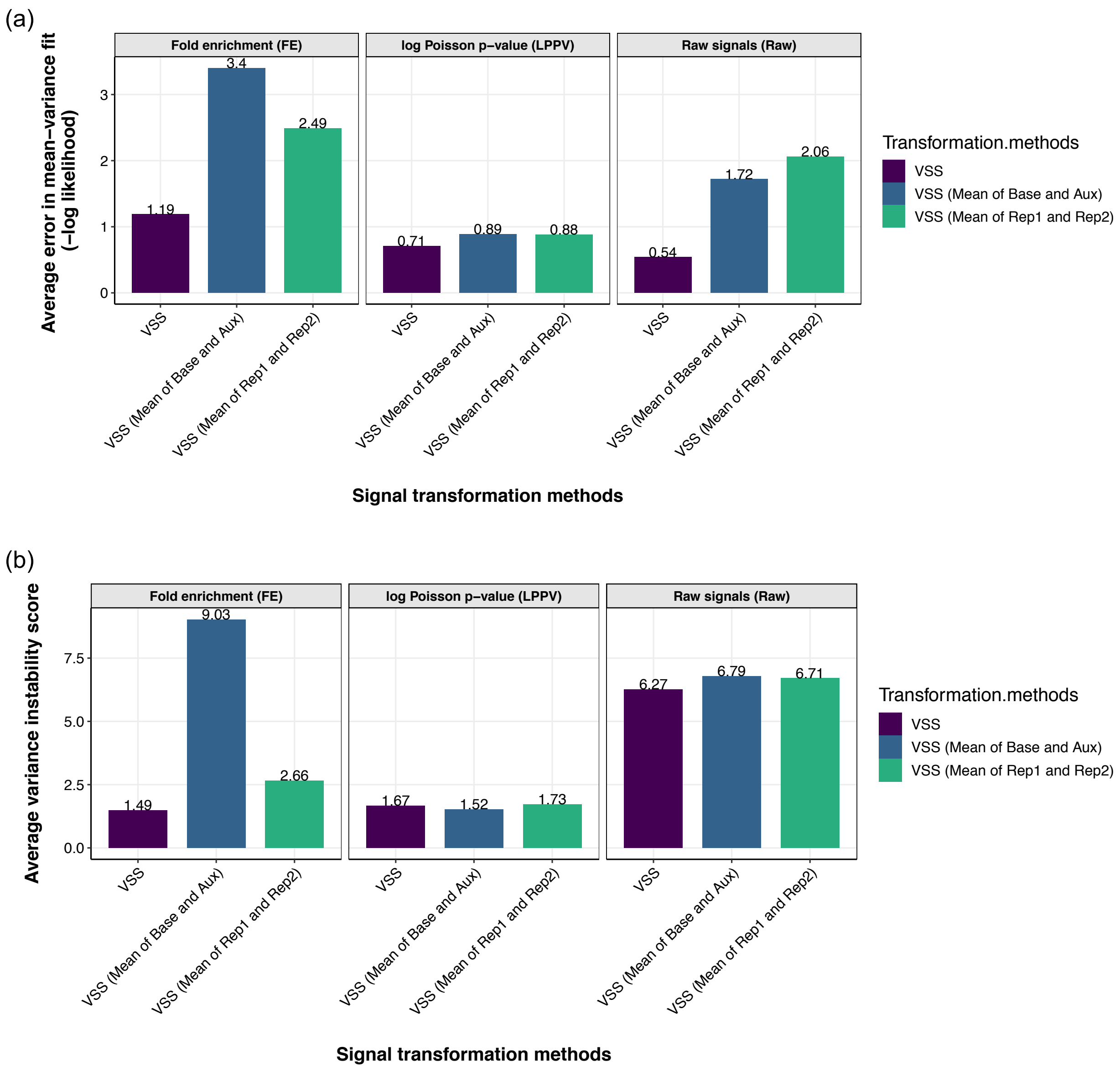

Fig. 6. Quantifying performance of alternative variations of VSS. (a) Goodness of fit to the mean-variance relationship derived from fold enrichment signals (FE), log Poisson p-value (LPPV) and raw signals (Raw), measured by Gaussian log likelihood (Methods). Lower values of negative log likelihood indicates better fit. Log likelihood was computed on chromosome 21; VSS's mean-variance relationship was trained on chromosome 22. (b) Variance instability score on fold enrichment signals (FE), log Poisson p-value (LPPV) and raw signals (Raw) (Methods). Lower values indicate more stable variance.

F High-quality vs low quality experiments

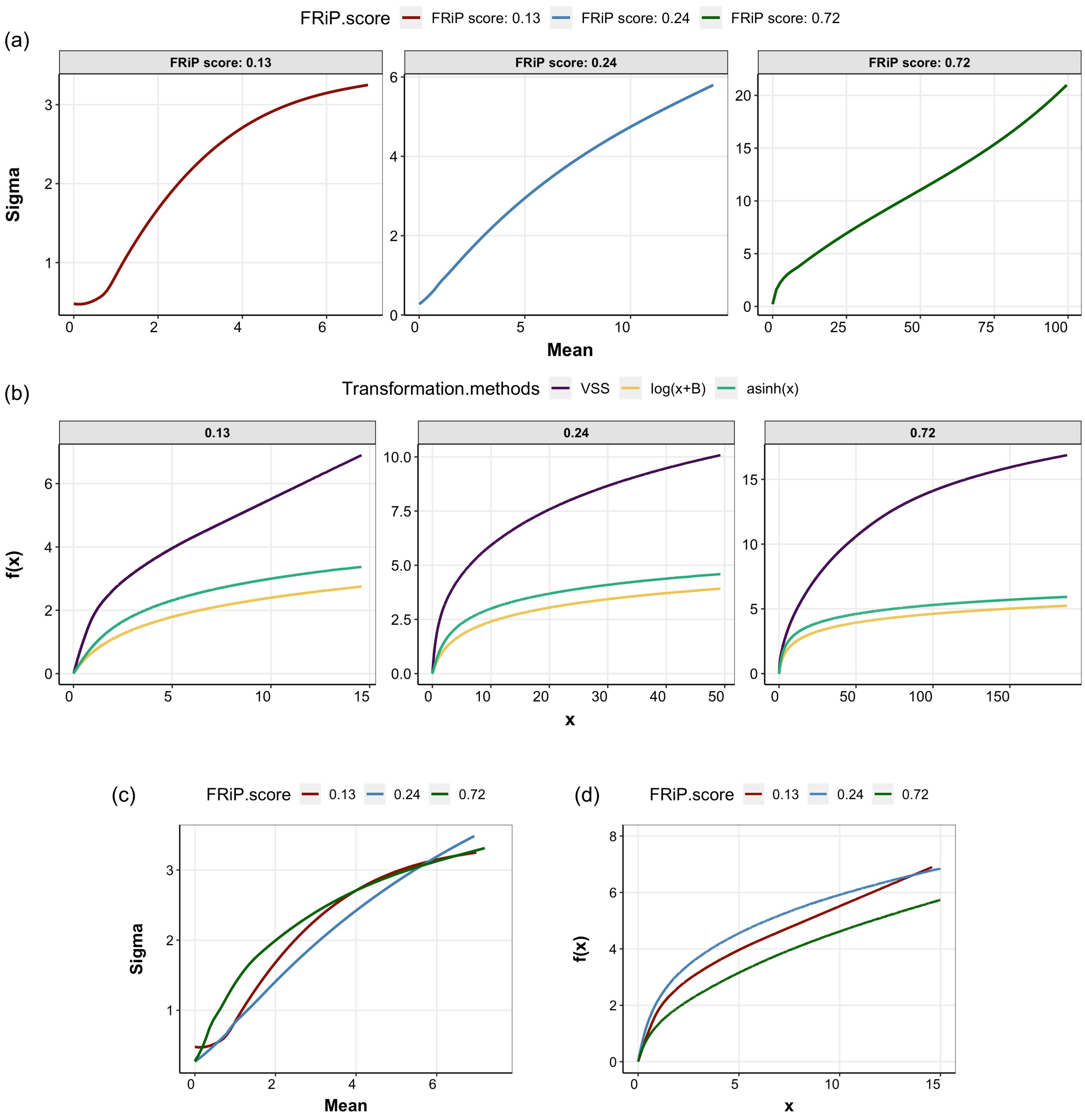

Fig. 7. Comparison between high quality experiments vs low quality experiments for H3k4me3 GM12878. Quality of experiments have been measured by FRiP score which quantifies the **fraction of reads in peaks**. a) mean-variance relationship differs between different qualities. b) indicates transformed signals for different FRiP scores. c) and d) same as a) and b) but in a limited range of signals.

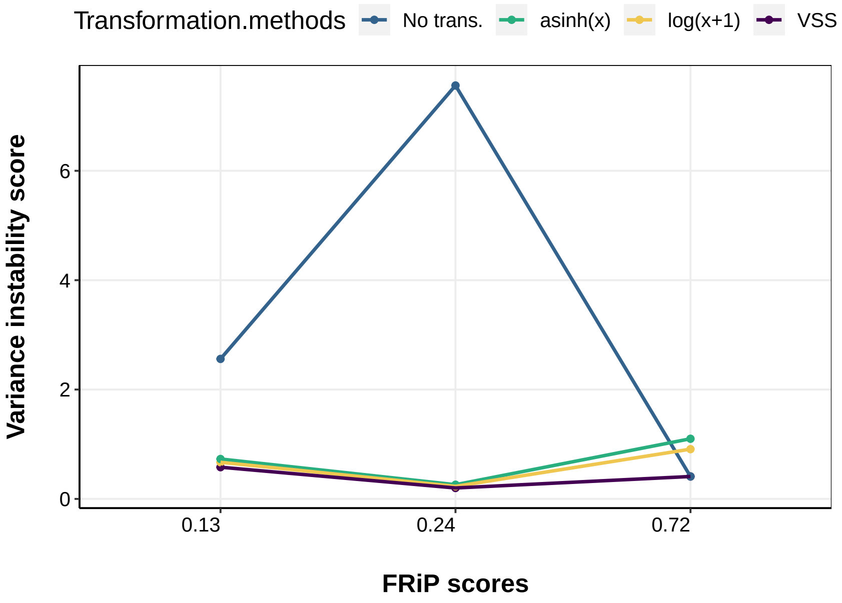

Fig. 8. Variance instability score for different peak qualities for Fold enrichment signals (FE) on H3k4me3 GM12878 (Methods). Horizontal axis indicates the FRiP score (**fraction of reads in peaks**) and vertical axis indicates the variance instability score. Lower values indicate more stable variance. For the experiment with high quality peaks, either $\log(x+1)$ or $\mathrm{asinh}(x)$ transformation have higher variance-instability score than the untransformed signals meaning that either of these transformations destabilize the variance. Results are shown in chromosome 21 for the VSS model trained on chromosome 22.

G Differences between replicates are stabilized after transformation

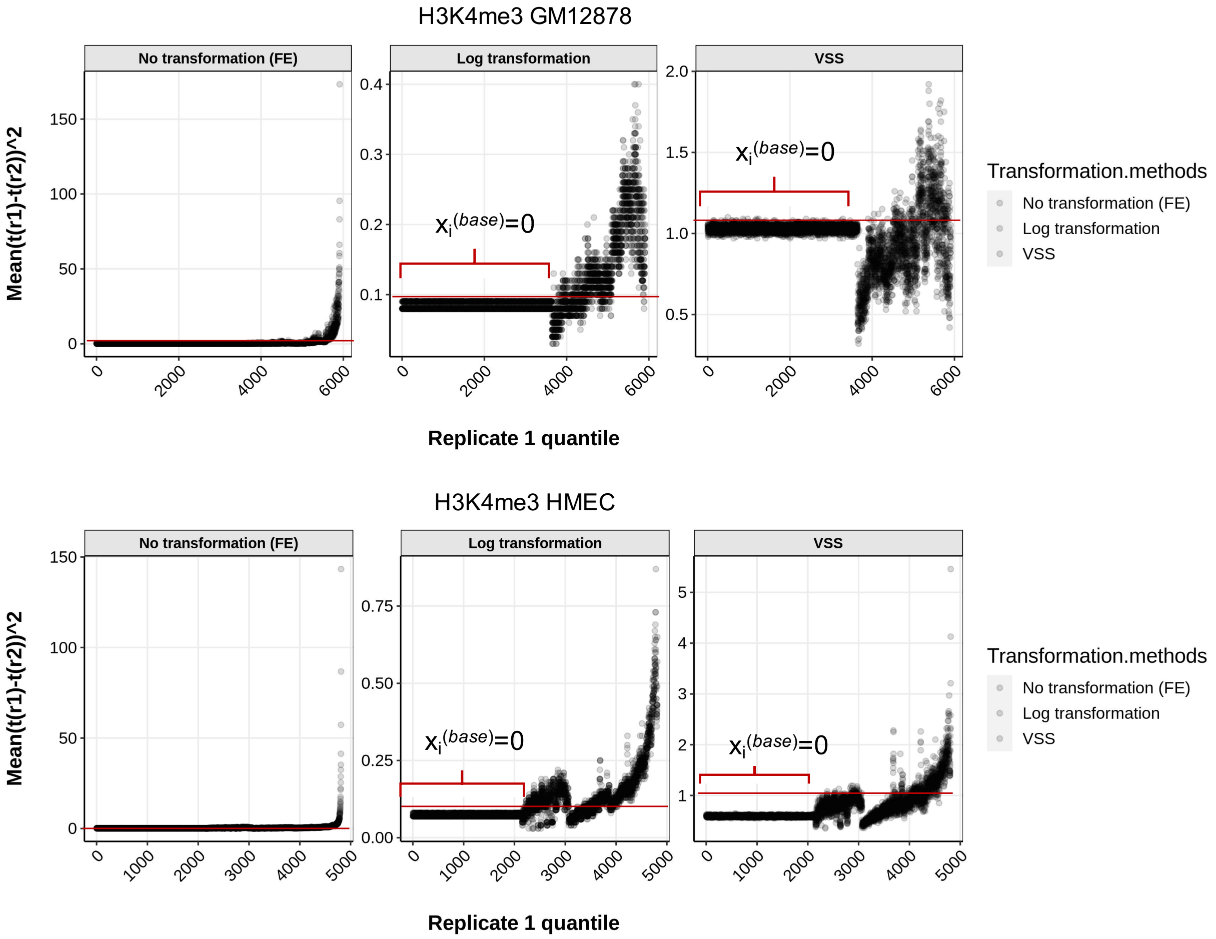

Fig. 9. Variance instability of transformed signals in different cell lines. Each point corresponds to a bin, where binning is defined according to replicate 1 value (Methods). Horizontal axis indicates binning index. Vertical axis indicates squared difference of values between replicates of an experiment. The flat line on the left half of each plot corresponds to positions where $x^{(\mathrm{base})} = 0$. Signals with stable variance show a flat (constant) trend on this plot; a trend (increasing or decreasing) indicates unstable variance. Note that VSS transformation has better (lower) variance instability than the untransformed signals (FE).

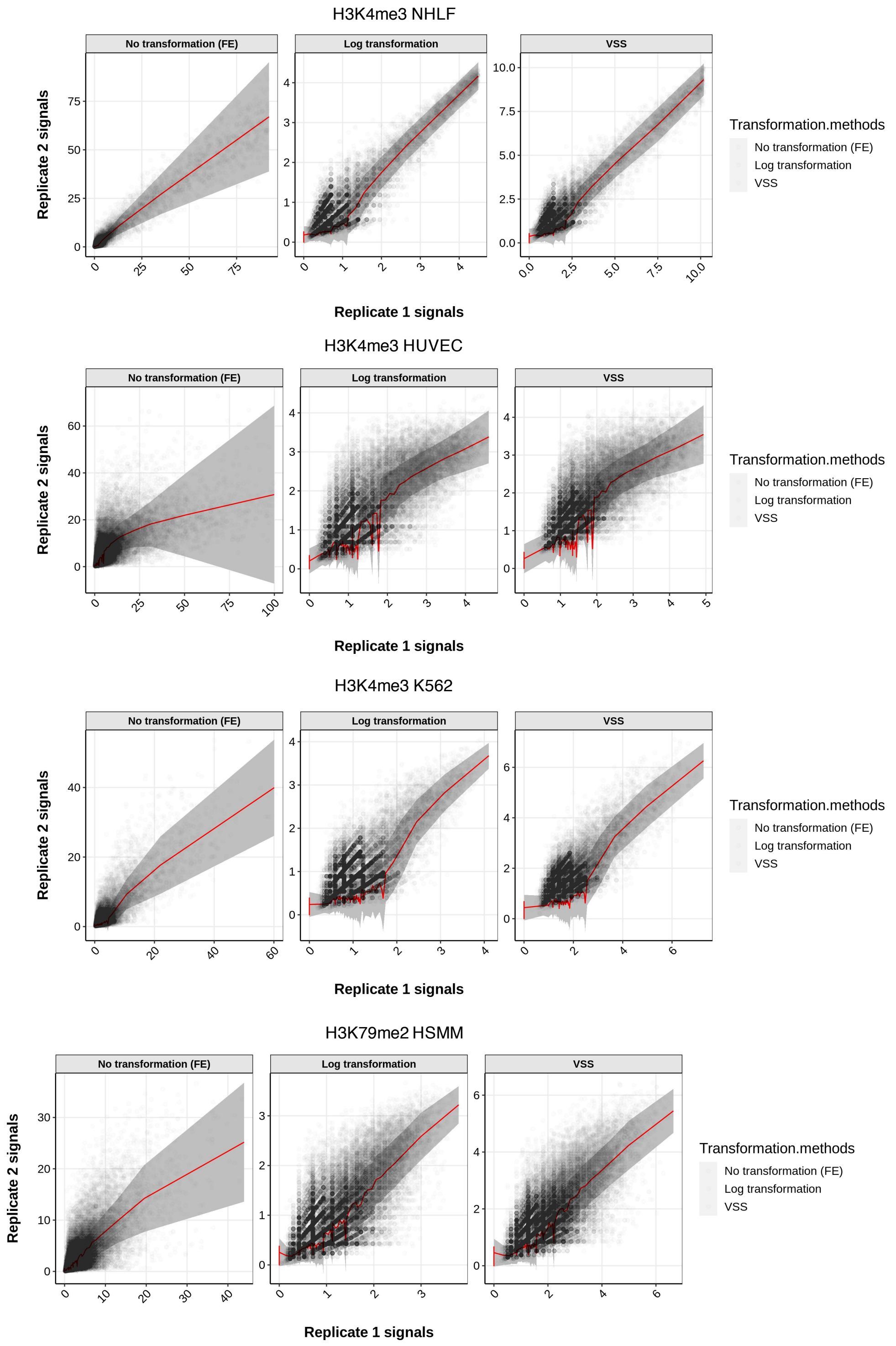

Fig. 10. Replicate 1 vs Replicate 2 signals before and after transformation in different cell lines. Shaded area represents the average variance in replicate 2 for a given value of replicate 1; variation in width of shaded region indicates a nonuniform mean-variance relationship. As shown in the plots, VSS and log transformation have stabilized the variance of the data while untransformed data has unstabilized signals. Note that results are shown in chromosome 21 for the VSS model trained on chromosome 22.

H VSS signals improve segmentation and genome annotation (SAGA) algorithm

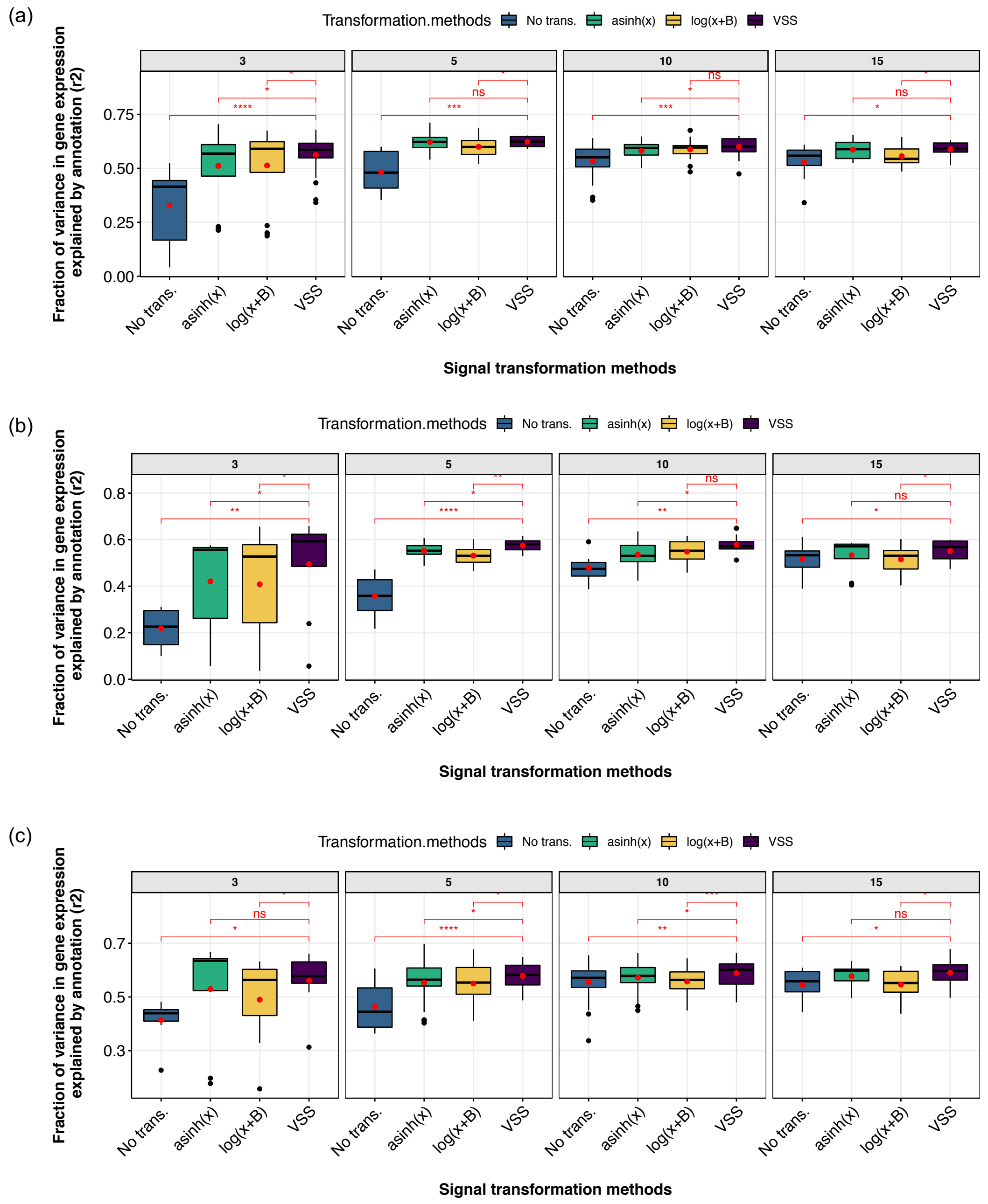

Fig. 11. Evaluation of annotations relative to gene expression. Vertical axis is the fraction of variance in gene expression explained (r2 , Genome annotation evaluation section). Horizontal axis are the transformation methods. Each panel represents number of features or states in a given model respectively (k) in (a) fold enrichment signals (FE) (b) log Poisson p-value (LPPV) (c) raw signals (Raw). Brackets indicate significance of VSS transformation to the other methods according to p-value from paired one-sided Wilcoxon signed rank test. Results are shown on chromosome 21 for an VSS model trained on chromosome 22.
